# The Industrial Sprawl in China from 2010 to 2019: A Multi-Level Spatial Analysis Based on Urban Scaling Law

**DOI:** 10.3390/ijerph192316255

**Published:** 2022-12-05

**Authors:** Lu Zhang, Xuehan Lin, Bingkui Qiu, Maomao Zhang, Qingsong He

**Affiliations:** 1 School of Public Administration, Central China Normal University, Wuhan 430079, China; 2 Department of Tourism Management, Jin Zhong University, Jinzhong 030619, China; 3 College of Public Administration, Huazhong University of Science and Technology, Wuhan 430074, China

**Keywords:** industrial sprawl, urban scaling law, spatial-temporal pattern, China

## Abstract

Studying the spatial-temporal distribution industrial sprawl in China is important to solve industrial sprawl problems and promote urban sustainable development. This paper constructed a multi-level spatial analysis of the Chinese industrial sprawl during 2010–2019 by mainly using urban scaling law, supplemented by GIS methods. Results showed that: (1) China had obvious industrial sprawl with a growth rate of 31.79%, reaching 2762.37 km^2^ between 2010 and 2019. (2) There was a stronger industrial sprawl in large cities with a larger population according to urban scaling law, especially in the East. (3) The industrial sprawl was mainly concentrated in the cities in the Northeast, Beijing-Tianjin-Hebei region, Shandong Peninsula, Yangtze River Delta region, Pearl River Delta region, Middle Yangtze River region, Fujian Province, and some cities in the West. (4) The gravity center of industrial sprawl generally moved southwest and distributed in Hubei Province. This study provided references for improving the efficiency of industrial land use and promoting high-quality urban development.

## 1. Introduction

Under the long-term process of global urbanization [1], urban sprawl has become a global phenomenon and challenged to sustainable development [2]. After the reform and opening up in 1978, China’s urbanization speed has led the world [3], resulting in rapid urban sprawl [4]. As two engines of economic development, industrialization and urbanization develop in close coordination and promote each other. China’s rapid urbanization has been accompanied by rapid industrialization [5], greatly promoting fast and massive industrial sprawl [6]. Thus, industrial land has become one of the most leading land use types in most Chinese cities [7].

Industrial development is very crucial for economic development [8], and industrial land is the most basic support for industrial development [9]. Thus, local governments encourage investment in the industrial sector and provide sufficient industrial land for industrial development to promote the development of the real economy [10]. For the past few decades, China’s local governments had promoted quick industrialization to boost local economic development through cheap industrial land and numerous industrial development zones [11,12], resulting in rapid industrial sprawl faster than urban expansion [13]. Since the 1990s, the number of industrial development zones in China has increased dramatically, greatly stimulating industrial sprawl [14]. In 2003, the Chinese government regulated and controlled the industrial development zones to curb its excessive growth and inefficient use, reducing its number by 70%. In addition, the Chinese government implemented the policy of controlling the minimum price for industrial land transfer to curb the inefficient use and idling of industrial land since 2006 [15]. However, as the world’s largest manufacturing and developing country [16], China required abundant industrial land and its industrial land still expanded quickly after, causing the proportion of industrial land in the urban area of China (20%) to be much higher than the world average level (10%) [17]. Moreover, industrial sprawl has widely appeared in rural areas as well as urban areas, resulting in deteriorative rural environment [18]. After decades of rapid industrialization, industrial sprawl in Chinese cities has triggered sustainable development problems such as disordered industrial expansion [19], environmental pollution [6], cultivated land loss [20], excessive carbon emissions [5], and inefficient land use [21]. Since 2014, the Chinese government has been promoting a new urbanization strategy to develop efficient and sustainable cities [22]. The 2030 Agenda for Sustainable Development of the UN in 2015 has proposed the goal of building sustainable cities [23]. Thus, it is necessary for the urban sustainable development to study the spatial-temporal pattern of industrial sprawl to optimize the spatial layout of industrial areas and realize intensive use of industrial land.

Urban sprawl has become a common phenomenon in global development, gaining great attention of many scholars [24]. Compared with studies on urban sprawl, the studies on industrial sprawl have not attained much attention. However, industrial sprawl has appeared in many developed and developing countries and has caused serious environmental pollution [6], attracting some scholars to focus on industrial sprawl. Many studies use remote sensing image or construct an evaluation index to quantitatively analyze the spatial-temporal pattern of industrial sprawl [14,25,26]. Moreover, some studies use regression models like OLS, the logistic regression model, the spatial econometric model, and the random forest model to analyze the relationships between industrial sprawl and industrial land marketization [27] and environmental securities [18], and to study the driving forces of industrial sprawl [28]. However, there is a lack of studies using innovative methods and perspectives to explore the spatial-temporal pattern of industrial sprawl and to provide suggestions for solving problems caused by industrial sprawl in the interest of achieving urban sustainable development.

Urban scaling law is a universal mechanism in urban systems providing a new idea of urban studies [29]. On the basis of previous studies, the scholar summarized urban scaling law and verified it via urban empirical data [30]. Since then, scholars proved that the urban scaling law can apply to various urban systems in different countries and regions [31,32,33]. Moreover, the urban scaling law applies not only to modern cities, but also to ancient cities and human settlement [34,35]. The urban scaling law is able to explain the non-linear relationship between urban indexes and population size, which has been widely used in urban studies [36]. Although some studies had analyzed Chinese cities from the perspective of scaling law [37], there are no studies using it to study China’s industrial sprawl.

To improve the efficiency of industrial land use and promote urban sustainable development, this paper applies urban scaling law to explore the spatial–temporal pattern of industrial sprawl in China using the data of industrial land from 2010 to 2019 of Chinese cities. As a supplement, traditional methods like kernel density estimation, cold–hot spot analysis, and a weighted standard deviation ellipse are also used to improve the comprehensiveness.

## 2. Materials and Methods

### 2.1. Study Area and Data Sources

**Figure 1:**
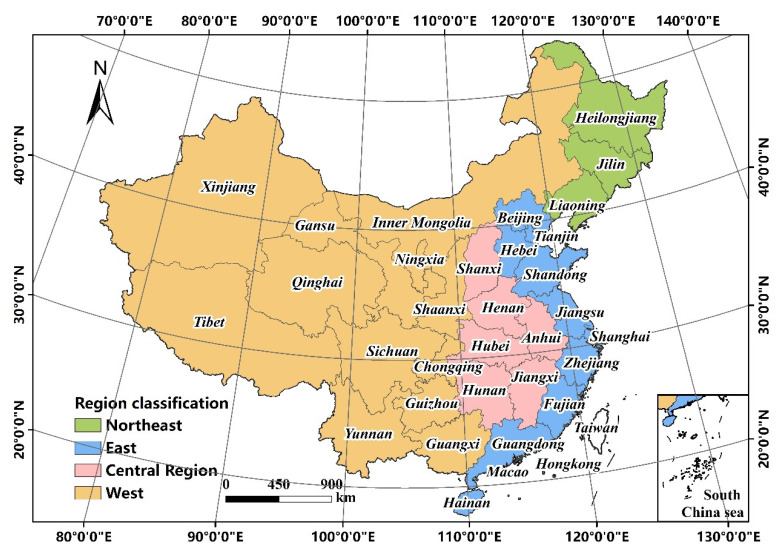
Research scope and regional classification.

China has 34 provincial-level administrative regions and is divided into four regions: the East, West, Northeast, and Central Region (Figure 1). Moreover, urban agglomerations have become important carriers of urban development and prefecture-level cities are the urban basic unit in China. Thus, this paper constructed the multi-level analytical framework of industrial sprawl containing the regional level, urban agglomeration level, and prefecture-level city level. In terms of data sources, this paper used data of urban permanent population and industrial land (excluding the data of Hong Kong, Macao and Taiwan). The data of the permanent population were obtained from the Statistical Yearbook of prefecture-level cities in China. The industrial land area was acquired from Statistical Yearbook of Urban Construction of China. Moreover, we obtained land transfer data of industrial land to analyze newly added industrial land from China Land Market official website (https://www.landchina.com (accessed on 14 November 2022)). The data include the information of location, transfer time, land area, industrial classification and so on. Furthermore, we used ArcGIS 10.8 to spatialize data by using the location information to prepare for further processing. Due to availability of data, our research period is during 2010–2019.

### 2.2. Methods

#### 2.2.1. SAMI of Urban Scaling Law

The Kleiber law indicates that there is a 3/4 power relationship between the size of an organism and its metabolic rate, called the scaling law [38]. The scaling law is not only a universal law existing in complex biology systems, but also a mechanism contained in urban systems. The formula is as follows:

(1) $$Y=Y_{0}N^{\beta }$$

where Y represents industrial land area in the paper; N is the urban permanent population; $Y_{0}$ and β are parameters, and β is the scaling exponent and coefficient of the scaling law. According to the relationship between β and 1, there are three conditions of urban scaling law: when β≈1, there is a linear relationship between industrial land area and permanent population; when β<1, their relationship is sublinear, indicating that from small cities to large cities, the growth rate of industrial land is smaller than the growth rate of permanent population, reflecting the scale economy of industrial land; when β>1, their relationship is superlinear, indicating that the growth rate of industrial land is greater than the growth rate of population from small cities to large cities, which reflects the effect of increasing returns to scale of industrial land.

Moreover, the scale-adjusted metropolitan indicator (SAMI) based on urban scaling law can eliminate population influence to compare cities of different sizes [39]. Thus, we used SAMI to compare the industrial sprawl of different cities. The formula is as follows:

(2) $$\log Y=\beta \times \log N+\log Y_{0}$$

(3) $$SAMI_{i}=\log Y_{i}-\log(Y_{0}N_{i}^{\beta })=\log(Y_{i}/Y_{0}N_{i}^{\beta })$$

where $SAMI_{i}$ represents the industrial land area that eliminates population influence; $Y_{i}$ is the true value of industrial land area; $Y_{0}N_{i}^{\beta }$ is its estimated value; $N_{i}$ is urban permanent population; β and $\log Y_{0}$ are fitting parameters.

#### 2.2.2. Kernel Density Estimation

As a widely used non-parametric estimation method in spatial analysis [40], kernel density estimation can visually show the spatial density of point data and effectively represent the spatial distribution of research objects. In this paper, kernel density estimation is used to describe the density of the spatial distribution of newly added industrial land points, and clearly measure the location and intensity of the spatial distribution of industrial sprawl in China. The kernel density estimation function formula is as follows:

(4) $$f(x)=\frac{1}{nh}\sum_{i=1}^{n}K(\frac{x-x_{i}}{h})$$

where f(x) represents the kernel density value of newly added industrial land points; K() is kernel function; h is the spatial range of newly added industrial land points, which is the bandwidth value; n is the number of newly added industrial land points in bandwidth value; $x-x_{i}$ is the distance between x and $x_{i}$.

#### 2.2.3. Cold–Hot Spot Analysis

The cold–hot spot analysis can identify the cluster of high value and cluster of low value of the elements on the space and is a good way to find spatial clustering characteristics [41]. This paper used cold–hot spot analysis to analyze the newly added industrial land area to further understand the spatial clustering characteristics of industrial land in China. The formula is as follows:

(5) $$G_{i}^{*}(d)=\sum_{j=1}^{n}W_{ij}(d)X_{i}X_{j}/\sum_{j=1}^{n}X_{j}$$

(6) $$Z(G_{i}^{*})=\frac{G_{i}^{*}-E(G_{i}^{*})}{\sqrt{Var(G_{i}^{*})}}$$

where $W_{ij}(d)$ is the spatial weight, the adjacent is 1 and the non-adjacent is 0; $X_{i}$ and $X_{j}$ are the observed values of region i and region j; $E(G_{i}^{*})$ and $Var(G_{i}^{*})$ are the expected value and variation of $G_{i}^{*}$, respectively. If $Z(G_{i}^{*})$ is positive and significant, it indicates that the newly added industrial land area around region i is higher than the mean value and region i belongs to the hot spot area. If $Z(G_{i}^{*})$ is negative and significant, region i belongs to the cold spot region.

#### 2.2.4. Weighted Standard Deviation Ellipse

The standard deviation ellipse can effectively and accurately reveal the overall characteristics of the spatial distribution of geographical elements [42]. We took the newly added industrial land area as weight to create standard deviation ellipses. The formula of its weighted mean center is as follows:

(7) $$\bar{X}=\sum_{j=1}^{n}w_{j}a_{j}/\sum_{j=1}^{n}w_{j};\bar{Y}=\sum_{j=1}^{n}w_{j}b_{j}/\sum_{j=1}^{n}w_{j}$$

where $\bar{X}$ and $\bar{Y}$ are the weighted average center; j is the data of sites of newly added industrial land area; n is the sum of sites of newly added industrial land area; $w_{j}$ is the area of the sites j of newly added industrial land area; $a_{j}$ and $b_{j}$ represent the longitude and latitude coordinates of the sites j of newly added industrial land area.

## 3. Results

### 3.1. Regional Difference of Industrial Sprawl

In order to compare the regional difference of industrial sprawl in China, this paper calculated the area of industrial land and its growth rate in the East, Central region, West, and Northeast for the years of 2010, 2013, 2016, and 2019. As shown in Table 1, the total area of the nationally industrial land increased from 8689.49 km^2^ to 11,451.86 km^2^ from 2010 to 2019, having a growth rate of 31.79%. Moreover, we also found nationwide per capita industrial land increases from 7.06 m^2^ to 8.94 m^2^ between 2010–2019. The results show industrial sprawl speed is quicker than the increasing speed of national population, showing that there is an obvious tendency of industrial sprawl in China. In terms of the growth rate of industrial land area in different periods, the nationwide growth rate of the 2013–2016 is the highest, reaching 15.04%, followed by growth rate of 2016–2019 (8.80%) and 2010–2013 (5.29%). Furthermore, the industrial land area in all regions grows rapidly during 2010–2019, and the East (36.31%) has the highest growth rate, followed by the West (29.95%), the Central region (26.02%), and the Northeast (25.45%). The East is the most developed region in China, so its industrial land is the most significant, reaching 5913.99 km^2^ in 2019, and has the most obvious sprawl tendency. With the implementation of China’s strategy of Western development, Central development, and Northeast revitalization, the West, Central region, and Northeast have experienced industrial development, resulting in their industrial sprawl.

**Figure 2:**
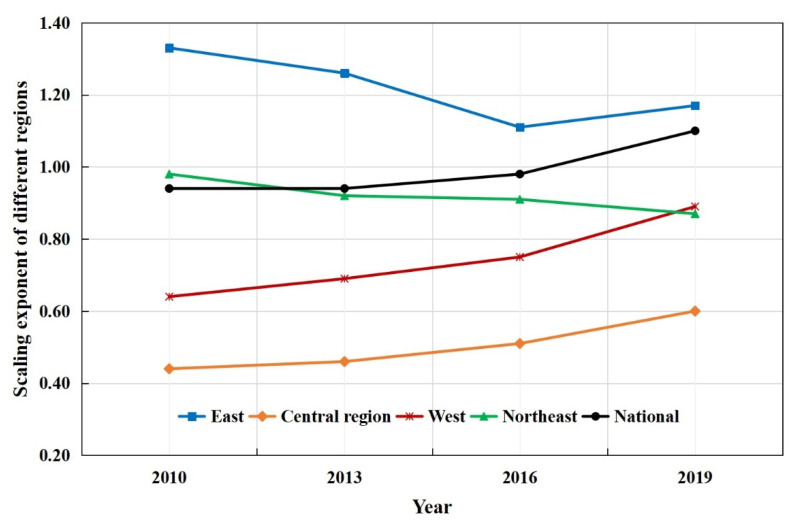
Scaling exponents of industrial land of different regions in China, 2010–2019.

In terms of scaling exponents of industrial land and permanent population, the results show that national scaling exponents constantly increase from 0.94 to 1.10, so the growth rate of industrial land is larger than the growth rate of the permanent population from smaller cities to larger cities in 2019 (Figure 2). Conversely, the growth rate of industrial land is smaller than the growth rate of the permanent population from smaller cities to larger cities in 2010, 2013, and 2016. Thus, the cities with a larger population have changed from having less per capita industrial land than the cities with a smaller population to having more per capita industrial land, indicating that f industrial sprawl is more obvious in the cities with a larger population in 2019 compared with 2010. Furthermore, the East has a larger scaling exponent (*β* > 1), indicating the effect of the increasing returns to scale of industrial land, and that the industrial sprawl is more prominent in the cities with larger population in the East. In addition, the Northeast region, West region, and Central region have smaller scaling exponents (*β* < 1), indicating the per capita industrial land is smaller and the scale economy of industrial land is more obvious in the cities with a larger population in these regions.

### 3.2. Difference of Urban Agglomerations of Industrial Sprawl

**Figure 3:**
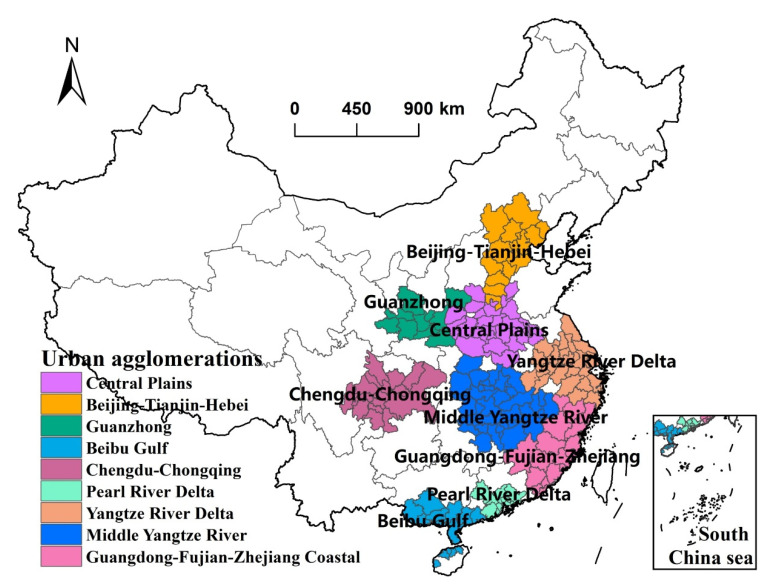
Nine national urban agglomerations in China.

According to data availability, this paper takes national urban night agglomerations as analysis units of urban scaling law to analyze differences of industrial sprawl in urban agglomeration level (Figure 3). According to results, scaling exponents of Beijing-Tianjin-Hebei, Yangtze River Delta, Chengdu-Chongqing, and Guanzhong shows the effect of increasing returns to the scale of industrial land, and the cities with a larger population have higher per capita industrial land in these regions (Table 2). On the contrary, we found that scaling exponents of the Pearl River Delta, Middle Yangtze River, Central Plains, Beibu Gulf, and Guangdong-Fujian-Zhejiang Coastal shows the scale economy of industrial land, and the cities with a larger population have lower per capita industrial land in these regions. Furthermore, the growth rate of industrial land exceeds that of permanent population from larger cities to smaller cities in Beijing-Tianjin-Hebei, Yangtze River Delta, Chengdu-Chongqing, and Guanzhong, indicating that the industrial land in these regions is concentrated in the core cities with a larger population and the agglomeration effect of the industrial economy. Therefore, cities with a larger population in Beijing-Tianjin-Hebei, Yangtze River Delta, Chengdu-Chongqing, and Guanzhong urban agglomeration have a more obvious trend of industrial sprawl.

### 3.3. Urban Difference of Industrial Sprawl

**Figure 4:**
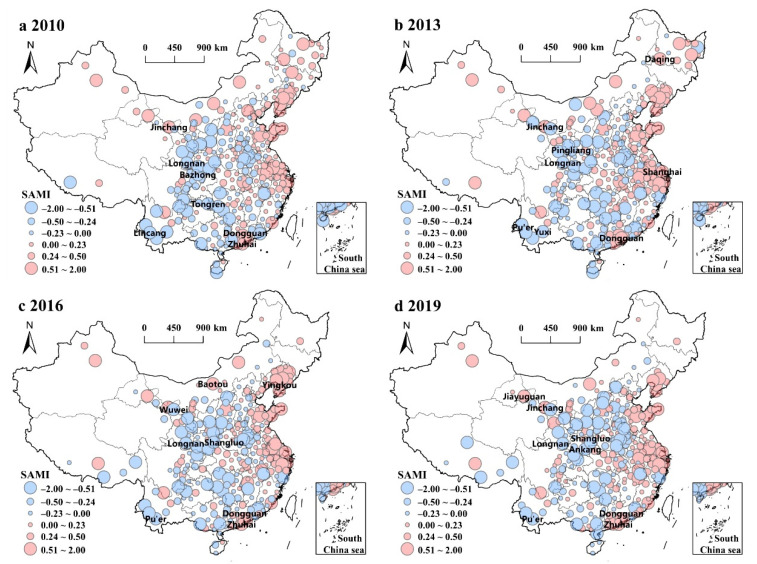
SAMI of urban scaling law of industrial land area in China in 2010–2019.

This paper used urban scaling law to obtain the SAMI of industrial land to eliminate the effect of urban population size, which can accurately analyze urban difference of industrial sprawl. The results show (Figure 4) that positive SAMI generally concentrates in the cities in the Northeast, Beijing-Tianjin-Hebei region, Shandong Peninsula, Yangtze River Delta region, Pearl River Delta region, Middle Yangtze River region, and Fujian Province. It shows the industrial land of most cities in these regions like Dongguan City, Shenzhen City, Zhuhai City, and Shanghai City is more than that of cities of same population scale, and there is an obvious situation of industrial sprawl in these regions. Moreover, the SAMI of some cities in the West is positive, such as some cities in the Chengdu-Chongqing urban agglomeration, Jiayuguan City, Jinchang City, and Baotou City, indicating that with the implementation of Western development strategy, some under-development cities in the West had experienced industrial sprawl due to cheap industrial land. Thus, these cities in the West have more industrial land than other cities with the same population. Furthermore, the negative SAMI mainly concentrates in the cities in the Central region and West of China except Middle Yangtze River region and Chengdu-Chongqing urban agglomeration, such as Longnan City, Pu’er City, Shangluo City, and Tongren City except. It shows that most cities in these regions have less industrial land compared to cities with same population size due to lagging economic levels, and their industrial sprawl is not obvious. In addition, the negative SAMI of −2.00~−0.51 moved from the Sichuan Province, Guizhou Province, and Yunnan Province in the southwest of China to Shanxi Province and Henan Province during 2010–2019, indicating the industrial sprawl of the Shanxi Province and Henan Province is obviously weakened.

**Figure 5:**
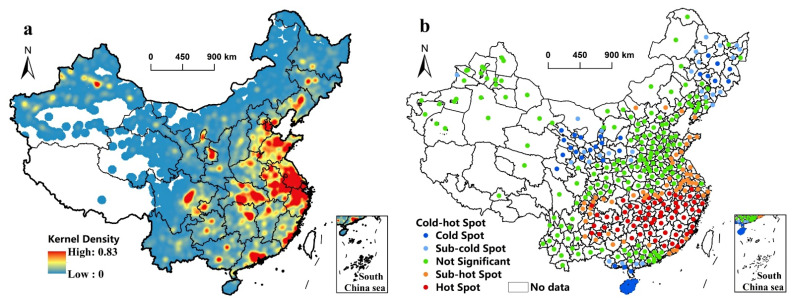
Kernel density estimation of newly added industrial land points from 2010 to 2019 in China (**a**); Cold–hot spot analysis of newly added industrial land area of prefecture-level cities from 2010 to 2019 in China (**b**).

We also analyzed the data of newly added industrial land points of China from 2010 to 2019 by kernel density estimation and cold–hot spot analysis to further explore the spatial aggregation characteristics of industrial sprawl in China (Figure 5). The results of kernel density estimation show that newly added industrial land points mainly gather around the Circum-Bohai Sea Region, Yangtze River Delta region, Middle Yangtze River region, the southeast coast of Fujian Province, Pearl River Delta region, Chengdu City, and Chongqing City from 2010 to 2019 in China. The cities in these regions have a higher kernel density value reaching 0.83 or nearly reaching 0.83, which means 0.83 newly added industrial land points per square kilometer. Thus, the number of newly added industrial land points in these cities is significantly more than in other cities, indicating there is more obvious industrial sprawl in these cities. For example, the cities with the largest number of newly added industrial land points are Jingzhou City (5126), Hangzhou City (4634), Shanghai City (3376), Quanzhou City (2908), and Chongqing City (2818). Moreover, we used the Jenks method to divide the results of cold–hot spot analysis into hot spots (including sub-hot spots), insignificant spots and cold spots (including sub-cold spots). Their proportion in the total is, respectively, 33.06% (hot spots), 47.50% (insignificant spots), and 19.44% (cold spots). According to results, hot spots focus on the southeast of Shandong Peninsula, Chengdu-Chongqing region, Guizhou Province, the north of Guangxi Province, the Middle Yangtze River region, Fujian Province, and the Yangtze River Delta region during the research period, while cold spots are located in Hainan Province, Leizhou Peninsula, the southeast of Guangxi Province, Shaanxi Province, Gansu Province, Ningxia Province, Heilongjiang Province, and Jilin Province. Therefore, the industrial sprawl of cities in hot-spot areas like Hangzhou City, Fuzhou City, Changsha City, Nanchang City, and Chengdu City is stronger, and their newly added industrial land are more than that of cities in other areas. Furthermore, compared with national urban agglomerations like the Yangtze River Delta, Middle Yangtze River, and Chengdu-Chongqing, industrial land in Northeast, Beijing-Tianjin-Hebei, and Pearl River Delta urban agglomeration tends to be saturated, so their newly added industrial land is smaller and their industrial sprawl is not obvious. In addition, newly added industrial land number and area are not accordant in spatial distribution. The newly added industrial land number has higher kernel density in the Circum-Bohai Sea region and Pearl River Delta region, while these places do not have a hot-spot of newly added industrial land area.

### 3.4. Direction, Gravity Center, and Range Evolution of Industrial Sprawl

**Figure 6:**
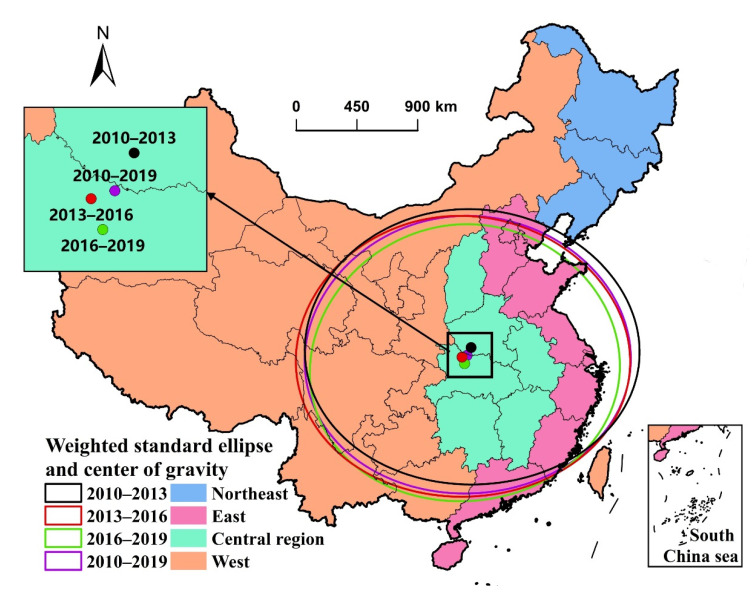
Weighted standard deviation ellipse of the industrial sprawl in China, 2010–2019 (The four colored dots express the gravity center of newly added industrial land area, and the four colored ellipses are the weighted standard deviation ellipses of 2010–2013, 2013–2016, 2016–2019, and 2010–2019).

This paper used results of a weighted standard deviation ellipse to analyze the evolution of distribution direction, gravity center, and distribution range of industrial sprawl (Figure 6 and Table 3). The gravity center can represent the spatial distribution center of industrial sprawl. The main direction of the spatial distribution of industrial sprawl can be reflected by the azimuth angle between the long axis and the north direction. By comparing the area of the ellipse, we can observe the change of spatial distribution range of industrial sprawl.

According to the movement of gravity center of industrial sprawl, compared with the gravity center of 2010–2013, the gravity center of 2013–2016 moved a large distance to the southwest, reaching 98.05 km. Compared with the center of gravity of 2013–2016, the gravity center of 2016–2019 moved a distance to the southeast, reaching 50.66 km. In conclusion, the gravity center of industrial sprawl in China during 2010–2019 experienced a process of moving from northeast to southwest and then to southwest, generally moving southwest, reaching a long distance of 128.38 km. Moreover, the gravity center during the research period was distributed in the Hubei Province located in the Middle Yangtze River.

In terms of the distribution range, the distribution range is expanded and then narrowed generally. Compared with 2010–2013, the distribution range of 2013–2016 is slightly narrowed and its growth rate is 1.21%. Moreover, the distribution range of 2016–2019 conspicuously reduces, and the ellipse area decreases by 9.04% and the semi-major axis (X StdDist) of the ellipse also decreases. In general, the distribution range of industrial sprawl from 2010 to 2019 mainly covers Central and Eastern China. It shows the industrial sprawl in the West is unapparent, and the newly added industrial land concentrates in the East and Central region of China.

According to the azimuth angle of the ellipse, there are some changes of distribution direction of industrial sprawl. The results show that compared with the ellipse of 2010–2013, the ellipse of 2013–2016 rotates anticlockwise by 0.54°; compared with the ellipse of 2013–2016, the ellipse of 2016–2019 rotates anticlockwise by 3.02°. Thus, the distribution direction generally rotates anticlockwise by 3.56° from 2010 to 2019, indicating the industrial sprawl in the East strengthens.

## 4. Discussion

After the tax distribution system reform in 1994, industrial land was the driving force for Chinese local governments to attract investment and drive economic growth. The government expropriated low-price land from rural collective economic organizations and released low-price industrial land to exchange for investment and future tax revenue [43], forming the “land-driven development” mode [27]. This mode denotes that China’s local governments control the land supply through their monopoly position in the land market and take land as an important tool for local governments to promote economic development and urbanization. A large amount of land is allocated for industrial use in various regions, which provides support for industrial development and generates industrial sprawl [44]. Thus, China has experienced industrial sprawl nationally, hindering the efficient use of industrial land and urban sustainable development [15]. Industrial land has increased by 2762.37 km^2^ in China, growing faster than the urban population during 2010–2019. Moreover, the industrial sprawl degree varies greatly in different regions and cities [45]. China’s industrial development showed a gradient trend—the industrial economy gradually declined from the East to the West. As the most economically developed area, the East has the highest growth rate of 36.31% of industrial land and the effect of increasing returns to scale of industrial land, showing the most obvious industrial sprawl during the research period. Most cities in the Beijing-Tianjin-Hebei region, Shandong Peninsula, Yangtze River Delta region, Pearl River Delta region, and Fujian Province had more industrial land than cities with same population. These regions are located in the East. In response to the strategy of Western development and Central development, the Chinese central government encouraged enterprises to invest in the West and Central region. After 2003, constructed land indexes (constructed land quotas assigned by central government to local governments) were used as a policy tool to give more industrial land to encourage industrial development in Central and Western China. Encouraged by the encouragement policies and industrial transfer from the East [46], the governments of the West and Central region have built many industrial parks to attract enterprises [11], which partly promotes the industrial development of these regions. Moreover, under the background of the Silk Road Economic Belt, the West and Central region have received a number of industrial transfers from abroad, stimulating their industrial sprawl. However, due to the lack of geographical advantages and economic basis, the industrial parks of these regions lacked competitive advantage, often accompanied by large amounts of idle land and huge environmental risks [21,47], which usually affects the ecological environment of the river downstream in the East [48].

Although industrial sprawl has contributed greatly to urban expansion and economic development in China [49], it is charactered by high pollution [18], high carbon emissions [50], and inefficient use [21], and thus industrial sprawl should be addressed to improve environment and urban sustainable development. The fiscal relationship between central and local governments should be reformed to avoid the excessive reliance of local governments on land for development. Moreover, the government should set minimum prices of industrial land in accordance with local conditions and strictly adhere to them [15], guiding the transfer of eastern industries to the West and Central region, and promoting industrial optimization and the upgrading and intensive use of industrial land in the East. Furthermore, the local government should rationally set up industrial development zones according to the economic development and population growth [13], restrain the speculation of industrial land, and punish the idle behavior of industrial land, promoting the intensive use of industrial land. In terms of urban planning, it should use scientific and reasonable urban planning to avoid the disorderly growth of industrial land, and set up an ecological red line to avoid occupying ecologically sensitive regions, especially in the West, Central region, and rural areas, causing a negative impact on the environment [18]. In reference to the successful experience of urban renewal of the “three old ” reconstruction in Guangdong Province [51], it is necessary for urban managers to use urban planning to redevelop the existing inefficient or idle industrial land rather than developing new industrial estates, gradually exploring the reduction in inefficient industrial land management to optimize the land-use pattern, promote industrial upgrading, and generate high-quality urban development in the East and large cities.

In summary, this paper analyzed the spatial-temporal evolution of industrial sprawl in China and summarized the evolution characteristics and connotations of industrial sprawl in various regions, urban agglomerations, and cities. However, this paper only makes a preliminary study combining the urban scaling law and industrial sprawl at the national scale. The research showed there is an urban scaling law in the intra-urban districts [37], which indicates the possibility of the study of industrial sprawl in a smaller scale, like a city. How to use scaling law to construct a systematic approach to explore small-scale industrial sprawl still need to be further explored.

## 5. Conclusions

The serious problems caused by industrial sprawl have exacerbated the unsustainability of urban development in the economy, society, and environment [52,53]. With the Sustainable Development Goals of the UN, it is necessary for us to establish a sustainability evaluation method for industrial sprawl. According to the objective law of global population urbanization process, we can apply urban scaling laws based on local population to appraise industrial sprawl. Thus, this paper developed an analytical framework of an urban scaling law method to quantitatively study the spatial-temporal pattern of China’s industrial sprawl during 2010–2019. We designed a multi-level spatial analysis based on the regional level, urban agglomeration level, and prefecture-level city level to comprehensively evaluate industrial sprawl. We found that: China’s industrial sprawl is obvious, and its industrial land increased by 31.79% during the research period, and the East showed the most prominent industrial sprawl. Moreover, there is stronger industrial sprawl in cities with a larger population in China. Furthermore, industrial sprawl in the Northeast, Beijing-Tianjin-Hebei, and Pearl River Delta urban agglomeration is not obvious compared with other urban agglomerations. Sustainable development cannot be achieved without significant changes in the way that urban space like industrial land is constructed and managed. In response to Sustainable Development Goals, this study is expected to measure industrial sprawl by the scaling law supplemented by GIS methods to provide scientific policy implications to solve industrial sprawl problems for establishing sustainable cities.

## Figures and Tables

**Table 1:** Regional difference of area and growth rate of industrial land in China, 2010–2019.

Region	Industrial Land Area (Unit: km^2^)				Growth Rate of Industrial Land			
	2010	2013	2016	2019	2010–2013	2013–2016	2016–2019	2010–2019
East	4338.77	4635.52	5572.06	5913.99	6.84%	20.20%	6.14%	36.31%
Central region	1703.36	1777.67	1812.56	2146.49	4.36%	1.96%	18.42%	26.02%
West	1558.94	1583.71	1796.67	2025.92	1.59%	13.45%	12.76%	29.95%
Northeast	1088.42	1152.68	1343.95	1365.46	5.90%	16.59%	1.60%	25.45%
National scale	8689.49	9149.58	10,525.24	11,451.86	5.29%	15.04%	8.80%	31.79%

**Table 2:** Scaling exponents of industrial land of night t urban agglomerations in China, 2010–2019.

Urban Agglomeration	2010	2013	2016	2019
Beijing-Tianjin-Hebei	1.68	1.63	1.45	1.78
Yangtze River Delta	1.11	1.24	1.38	1.29
Pearl River Delta	0.95	1.38	0.84	0.79
Chengdu-Chongqing	1.4	1.37	1.24	1.25
Middle Yangtze River	0.62	0.53	0.51	0.74
Central Plains	0.29	0.41	0.52	0.4
Guanzhong	1.15	1.27	1.00	1.15
Beibu Gulf	1.01	1.00	0.76	0.51
Guangdong-Fujian-Zhejiang	0.45	0.11	0.18	0.24

**Table 3:** Parameters of weighted standard deviation ellipse of industrial sprawl.

Year	X StdDist	Y StdDist	Area	Azimuth Angle	Center X	Center Y
2010–2013	13.43	9.10	383.92	92.69	112.31	32.89
2013–2016	13.32	9.29	388.56	92.15	111.53	32.30
2016–2019	12.30	9.14	353.42	89.13	111.70	31.87
2010–2019	13.01	9.17	375.03	91.18	111.94	32.39

## Data Availability

The data that support the findings of this study are available from the corresponding author upon reasonable request.
